# Prevalence of post COVID-19 condition and associations with risk factors among U.S. adults: 2023 Behavioral Risk Factor Surveillance System

**DOI:** 10.3389/fpubh.2025.1662273

**Published:** 2025-09-25

**Authors:** Gregory W. Heath, David Levine, Gloria Oppong, Majdi Alghader

**Affiliations:** ^1^Department of Health and Human Performance, College of Health, Education, and Professional Studies, University of Tennessee at Chattanooga, Chattanooga, TN, United States; ^2^Department of Internal Medicine, University of Tennessee Health Science Center College of Medicine, Chattanooga, TN, United States; ^3^Department of Physical Therapy, University of Tennessee at Chattanooga, Chattanooga, TN, United States; ^4^Department of Mathematics, University of Tennessee at Chattanooga, Chattanooga, TN, United States; ^5^Office of Research Integrity, University of Tennessee at Chattanooga, Chattanooga, TN, United States

**Keywords:** COVID-19, SARS-CoV-2, long-COVID, post COVID-19 condition, physical activity, chronic health conditions, behavioral risk factors, non-communicable diseases

## Abstract

**Introduction:**

During the COVID-19 pandemic, between 12 and 20% of US adults were identified as having post-COVID-19 condition, commonly referred to as ‘Long COVID’. These individuals maintained symptoms of COVID-19 for 3 months or longer following their illness but lacked an active infection. Using the Center for Disease Control’s 2023 Behavioral Risk Factor Surveillance System, our hypotheses were that adults who did not meet the 2018 Physical Activity Guidelines for Americans for aerobic and strengthening activities, those not fully vaccinated against COVID-19, and those with certain non-communicable diseases would be at greater odds of reporting post COVID-19 conditions.

**Methods:**

The association of post COVID-19 conditions were examined among the 46.4% of adults 18 years and older who had tested positive for COVID-19 (*n* = 201,248), with a subset these adults reporting post COVID-19 conditions (*n* = 27,074, 13.6%). Univariate and logistic regression analyses were conducted using SPSS (v29) for complex samples. Univariate analyses were initially conducted on both behavioral risk factors and multiple non-communicable diseases. Subsequently, a series of logistic regression analyses controlling for age, sex, race/ethnicity, and educational attainment were carried out to compare the outcome variable of post-COVID-19 conditions with the exposure variables of (1) not meeting the Physical Activity Guidelines for Americans, (2) not being fully vaccinated, or (3) having the non-communicable diseases of overweight/obesity, coronary heart disease, asthma, or hypertension.

**Results:**

Adults (*n* = 13,449; 12.2%) who did not meet the Physical Activity Guidelines for Americans were at greater odds of reporting post COVID-19 conditions (aerobic activity – OR = 1.19, 95% CI 1.06, 1.33, *p* < 0.0001; strengthening activity – OR = 1.02, 95% CI 1.00, 1.03, *p* < 0.001) compared with those meeting the guidelines. Respondents who were not fully vaccinated (≤ 3 vaccinations) were at greater odds of reporting post COVID-19 conditions (OR = 1.42, 95% CI, 1.24, 1.49, *p* < 0.0001) compared with those reporting ≥4 vaccinations.

**Discussion:**

The present findings support the hypothesis that adults who were female, did not achieve the Physical Activity Guidelines, were not fully vaccinated, and had certain non-communicable diseases demonstrated a stronger association with reporting post COVID-19 conditions following COVID-19 infection.

## Introduction

During the COVID-19 pandemic, 12–20% of US adults developed symptoms that persisted for 3 months or longer after their initial infection. Such people with lingering symptoms were diagnosed with post COVID-19 condition, more commonly referred to as ‘Long COVID’ (LC) (1). LC, which has also been called chronic COVID, long-haul COVID, and post-COVID, is defined as residual health problems beginning within 3 months and continuing for at least 2 months subsequent to an acute infection with the SARS-CoV-2 virus (2–6).

It is estimated that as many as 65 million individuals around the world have had LC, based on a conservative estimated incidence of 10% of infected people and more than 651 million documented COVID-19 cases worldwide; the number is likely much higher due to many undocumented infections. The incidence of LC is estimated at 10–30% of non-hospitalized infections, 50–70% of hospitalized cases (7, 8), and 10–12% of vaccinated cases. LC has been associated with all ages and acute phase disease severities, with the highest percentage of diagnoses between the ages of 36 and 50 years. Most LC infections are among non-hospitalized patients with a mild acute illness (9), as this population represents many overall COVID-19 infections (10).

### Background

The global prevalence of LC in a recent meta-analysis of 41 studies reported an estimated pooled prevalence of 43%. It was higher among patients who were hospitalized for COVID (54%) compared to non-hospitalized COVID patients (34%) (7).

In another large systematic review of 144 studies, the global estimated pooled prevalence of LC was 36% among COVID-19 positive individuals. Geographical variation was observed in the estimated pooled prevalence of LC: South America at 51%, Europe at 39%, Asia at 35%, and North America at 30% (11).

The five most prevalent LC subtypes among COVID-19–positive cases were respiratory (20%, 31 studies), general fatigue (20%, 121 studies), psychological (18%, 10 studies), neurological (16%, 23 studies), and dermatological (12%, 10 studies) (12). The most common symptom based on estimated prevalence was memory problems, estimated at 11% meta-analyzed from 12 studies (12). The three strongest risk factors for LC were being unvaccinated for COVID-19, pre-existing comorbidity, and female sex. Individuals with any of these risk factors had higher odds of having LC, with pooled estimated odds ratios of 2.34 (95% CI 1.49–3.67) meta-analyzed from six studies, 1.59 (95% CI 1.28–1.97) from 13 studies, and 1.55 (95% CI 1.25–1.92) from 22 studies (11).

In a systematic review of 57 studies with 250,351 survivors of COVID-19, the median (IQR) proportion of COVID-19 survivors experiencing at least one post-acute sequelae of SARS-CoV-2 infection (PASC) was 54.0% (45.0–69.0%; 13 studies) at 1 month (short-term), 55.0% (34.8–65.5%; 38 studies) at 2–5 months (intermediate-term), and 54.0% (31.0–67.0%; 9 studies) at six or more months (long-term) (13). The most prevalent were pulmonary sequelae, neurologic disorders, mental health disorders, functional mobility impairments, and general and constitutional symptoms such as chest imaging abnormalities (median [IQR], 62.2% [45.8–76.5%]), difficulty concentrating (median [IQR], 23.8% [20.4–25.9%]), generalized anxiety disorder (median [IQR], 29.6% [14.0–44.0%]), general functional impairments (median [IQR], 44.0% [23.4–62.6%]), and fatigue or muscle weakness (median [IQR], 37.5% [25.4–54.5%]) (12). Other frequently reported symptoms included cardiac, dermatologic, digestive, and ear, nose, and throat disorders (13).

Female sex, household size (≥2), low financial security, a negative impact of the COVID-19 pandemic on occupation and work conditions, number of comorbidities (≥2), presence of respiratory disease, mental and sensory disorders, number of SARS-CoV-2 infections (≥2), initial symptoms (≥6), and perceived high severity of COVID-19 are positively and consistently associated with long COVID (14). Age ≥ 75 years, retirement, SARS-CoV-2 vaccination (≥2 doses), and good perceived information regarding long COVID are negatively associated with the condition (5, 12, 14). A meta-analysis among adults who were hospitalized for COVID-19 from Iran, Russia, and Sweden revealed a prevalence of 29.19% for LC (7). Among children and adolescents, a meta-analysis consisting of 21 studies with 80,071 patients from Europe, Iran, Brazil, and Australia reported the overall prevalence of LC was 25.24% (8).

A systematic review and meta-analysis by Razak et al. estimated that the pooled prevalence of long COVID worldwide is 41.79% (15). Prevalence was estimated at 52.77% in females and 47.23% in males. For prevalence among continents, Asia was the highest with 49.79%, followed by America at 46.29%, Europe at 46.28, and 42.41% at Australia. Only one study from Africa was included in this review, and it reported a prevalence of 50.33%. Most of the subgroups showed a significant heterogeneity level with I^2^ = 100%, *p* < 0.01 (15).

A meta-analysis by Healey et al. reported the most common signs and symptoms of long COVID (9). The most common signs and symptoms observed at any point of long COVID diagnosis among the sample size of 10,643 were fatigue (37%), dyspnea (21%), olfactory dysfunction (17%), myalgia (12%), cough (11%), and gustatory dysfunction (10%). Less common symptoms observed included headache (7%), diarrhea (5%), and chest pain (3%). A systematic review by Natarajan et al. showed that general symptoms of pain, fatigue, fever, hair falling out, rash, and weight loss pooled prevalence was 14.4% of the 11,598-person sample (16). Fatigue was the most reported symptom from the “general” category, with a 29.2% pooled prevalence. Muscle pain prevalence was 13.30%. The pooled prevalence of joint pain was 28.25% and hair loss was 20.29%. Neurological symptoms reported in the study included headache, cognitive impairment, and loss of some senses (smell, taste, and/or hearing). The most prevalent neurological symptom was cognitive impairment at 28.85%.

### Research objectives

Our study examined associations between physical inactivity, vaccination status, selected non-communicable diseases (NCDs) and chronic conditions, and long COVID (LC) among US adults aged 18 years and older with a history of COVID-19 using data from the CDC’s 2023 Behavioral Risk Factor Surveillance System (BRFSS) (17).

## Methods

The 2023 BRFSS data was used to examine the prevalence of associations with selected risk factors for LC among the 46.4% of adults 18 years and older who had tested positive for COVID-19 (*n* = 201,248) and a subset of these adults who reported having LC (*n* = 27,074, 13.6%).

The BRFSS is a phone-based (landline and cell phone) survey coordinated by the National Centers for Disease Control and Prevention’s (CDC) National Center for Chronic Disease Prevention and Health Promotion. Surveys are administered yearly in each state and US territory. Survey data is collected monthly to control seasonality effects. The sampling frame uses complex sampling procedures, resulting in state-specific estimates as well as the aggregated state and territory data that yield nationally representative estimates. The BRFSS surveys have collected core behavioral risk factors, preventive practice behaviors, chronic disease prevalence, demographic data, rotating/optional modules, and state-added questions since 1984 (18). The 2023 data (last full year of data collection to date) consist of over 433,000 respondents (17).

The BRFSS definition of LC for the current study represents respondents who answered affirmatively to the following questions: (1) “have you ever tested positive for COVID-19 (using a rapid point-of-care test, self-test, or laboratory test) or been told by a doctor or other health care provider that you have or had COVID-19?”; and (2) “do you currently have symptoms that have lasted 3 months or longer that you did not have prior to having coronavirus or COVID-19?.” In answering this question, if respondents asked for more clarification about symptoms, the interviewer read the respondent the following list: “These conditions could be tiredness or fatigue; difficulty thinking or concentrating or forgetfulness/memory problems (sometimes referred to as “brain fog”); difficulty breathing or shortness of breath; joint or muscle pain; fast-beating or pounding heart (also known as heart palpitations) or chest pain; dizziness on standing; menstrual changes; symptoms that get worse after physical or mental activities; or loss of taste or smell.” Respondents also answered a third question about whether their LC symptoms/signs (1) significantly limited their activities of daily living, (2) somewhat limited their activities of daily living, or (3) did not affect their activities of daily living.

Both univariate and logistic regression analyses were conducted using SPSS (v29) for complex samples. Our initial univariate analyses examined all potential risk factors and associations as gleaned from an online search in PubMed Central using key search terms such as post COVID-19 conditions; Long COVID; Long-haul COVID; Post-COVID-19 syndrome; Chronic COVID; Post-acute sequelae of SARS-CoV-2 infection (PASC); and Infection-associated chronic condition (IACC). Our literature search was updated through July 2025. Our significant univariate findings included associations between LC and (1) the chronic conditions of overweight/obesity, type 2 diabetes, hypertension, asthma, or previous myocardial infarction or coronary heart disease; (2) the health behavior of not having met the 2018 aerobic physical activity guidelines for Americans; (3) the preventive health practice of not being fully vaccinated against COVID-19 (≤3 vaccinations vs. 4 or more vaccinations); (4) demographic factors of age 18–64 years vs. 65 years and older, female sex, and high school graduate or less vs. college or technical school graduate. Subsequent analyses were conducted including these co-variates in a series of logistic regression analyses controlling for age, sex, race/ethnicity, and educational attainment. Odds ratios (ORs) with 95% confidence intervals (CIs), along with the Mantel–Haenszel chi-square statistic and probability estimates, were reported in the final logistic regression model.

We compared the outcome variable of LC with the exposure variables listed above of (1) not meeting the Physical Activity Guidelines (PAGs) for aerobic and strengthening activity and (2) having ≤3 vs. 4 or more COVID-19 vaccinations.

## Results

The major demographic characteristics of respondents who reported having COVID-19, with and without LC, are presented in Table 1. The majority of those identified as having COVID-19 and LC were in the age range of 18–64 years, with most respondents being female. The major race/ethnic groups of White/Caucasian, Black/African American, and Hispanic/Latino-Latina are represented in descending order from the largest population.

**Table 1:** Demographic characteristics among U.S. adults 18 years and older with a history of COVID-19 who report having or not having long COVID: BRFSS, 2023.

Characteristic	Positive for long COVID *n* (%)	Negative for long COVID *n* (%)	Total *N*
Age 18–64 years	19,254 (14.2)	115,925 (85.8)	135,179
Age 65 years +	7,505 (12.5)	52,714 (87.5)	60,219
Female	17,292 (16.1)	90,474 (83.9)	107,766
Male	9,782 (10.8)	80,419 (89.2)	90,201
White/Caucasian	19,440 (13.3)	127,127 (86.7)	146,567
Black/African American	1,727 (14.2)	10,414 (85.8)	12,141
Hispanic/Latino-Latina	3,038 (15.4)	16,671 (84.6)	19,709
Not High School Grad	1,473 (18.2)	6,607 (81.8)	8,080
High School Graduate	6,470 (15.2)	36,162 (84.8)	42,642
Some College/Tech School	8,512 (16.4)	43,495 (83.6)	52,007
College or Tech Grad	10,531 (11.1)	84,103 (88.9)	94,634

Educational attainment represents a surrogate measure of socioeconomic status, with those having graduated from college or technical/vocational school representing the largest group of respondents (19).

The prevalence estimates among those variables identified as having a significant association with LC through logistic regression for complex samples are listed in Table 2. These variables included the estimates among those reporting having had COVID-19 as the denominator for the rates among those with LC. The significant associations identified included (1) not meeting the PAGs for aerobic activity and strengthening activity among respondents; (2) those respondents who were not fully vaccinated for COVID-19, i.e., having ≤3 doses of the COVID-19 vaccine compared with those who were considered ‘fully’ vaccinated, i.e., with 4 or more doses of the COVID-19 vaccine; (3) respondents who reported having a history of coronary heart disease; (4) respondents reporting to be either overweight or obese; (5) those reporting to have current asthma; and (6) those reporting to currently have hypertension.

**Table 2:** Prevalence estimates for not meeting the physical activity guidelines (aerobic and strength), vaccination status, and selected NCD among US adults 18 years and older with a history of COVID-19 who report having and not having long COVID: 2023 BRFSS.

Characteristic	Positive for long COVID *n* (%)	Negative for long COVID *n* (%)	Total *N*
Not meeting physical activity guidelines (aerobic activity)	9809/23,258 (42.2)	51,729/148,176 (34.9)	171,404
Not meeting physical activity guidelines (strengthening activity)	9,651/23,366 (36.6)	67,712/166,605 (41)	189,971
<3 COVID-19 vaccinations	5,092 (13.8)	31,813 (86.2)	36,905
4 or more COVID-19 vaccinations	1,894 (10.4)	16,290 (89.6)	18,104
History of coronary heart disease	2,921/27,661 (10.9)	11,601/169,710 (6.8)	196,401
Overweight or obese	19,003 (74.8)	112,119 (69.6)	186,509
Currently have asthma	5,279/26,744 (19.7)	17,087/169,666 (10.1)	196,410
Currently have hypertension	11,963/26,990 (44.3)	63,549/170,413 (37.3)	197,403

The final model in the logistic regression included the following: Dependent Variable: Do You Currently Have COVID-19 Symptoms? (reference category = No); Model: (Intercept): Factors and co-variates used in the computations: number of COVID Vaccinations; overweight or obese; currently have asthma; currently have hypertension; history of coronary heart disease; not meeting the physical activity guidelines for aerobic and strength physical activity (0–149 min of physical activity per week; race/ethnicity = 1.94; male or female sex = 1.54; Reported Age in two age groups = 1.22; Level of education completed CA = 3.05).

As reported in Table 3, LC demonstrated a strong association with several selected risk factors, both modifiable and non-modifiable. Respondents with COVID-19 who did not meet either the aerobic or strengthening Physical Activity Guidelines (PAGs) had significantly greater odds of reporting LC than those who met the guidelines Being fully vaccinated (four or more COVID-19 doses) appeared to be protective against LC, with those reporting not being fully vaccinated (≤3 doses) at significantly greater odds of LC compared to those who were fully vaccinated.

**Table 3:** Reported odds ratios and 95% confidence intervals for associations among those not meeting the physical activity guidelines (aerobic and strength), number of COVID-19 vaccinations, and selected chronic conditions with having “Long COVID.”

Variable	Odds ratio	95% Confidence interval	*p* value Chi-square
Did not meet physical activity guidelines – aerobic	1.19	1.06, 1.33	*p* < 0.001
Did not meet physical activity guidelines – strength	1.02	1.002, 1.02	*p* < 0.001
Not fully vaccinated (<3 COVID-19 vaccinations)	1.415	1.243, 1.492	*p* < 0.001
History of coronary heart disease	2.02	1.82, 2.24	*p* < 0.001
Overweight or obese	1.29	1.13, 1.47	*p* < 0.001
Currently have asthma	1.87	1.62, 2.17	*p* < 0.001
Currently have hypertension	1.37	1.22, 1.54	*p* < 0.0001
Female	1.59	1.42, 1.78	*p* < 0.0001
65 years old or older	0.76	0.67, 0.87	p < 0.001
College or technical school graduate	0.87	0.81, 0.92	*p* < 0.001

Current asthma, hypertension, coronary heart disease, and being overweight or obese were all significantly associated with LC, with coronary heart disease having an Odds Ratio of 2.02 (95% CI = 1.82, 2.24), demonstrating the strongest association. This finding supports the growing body of evidence showing a relationship between LC symptoms and a history of having selected NCDs and other chronic underlying conditions (20).

Additionally, the demographic risk factors of being female and less than 65 years old demonstrated higher odds of reporting LC. Conversely, those reporting that they were graduates of a technical or college program were at lesser odds of having LC. These findings concur with findings recently reported by CDC and other investigators (21, 22).

**Figure 1:**
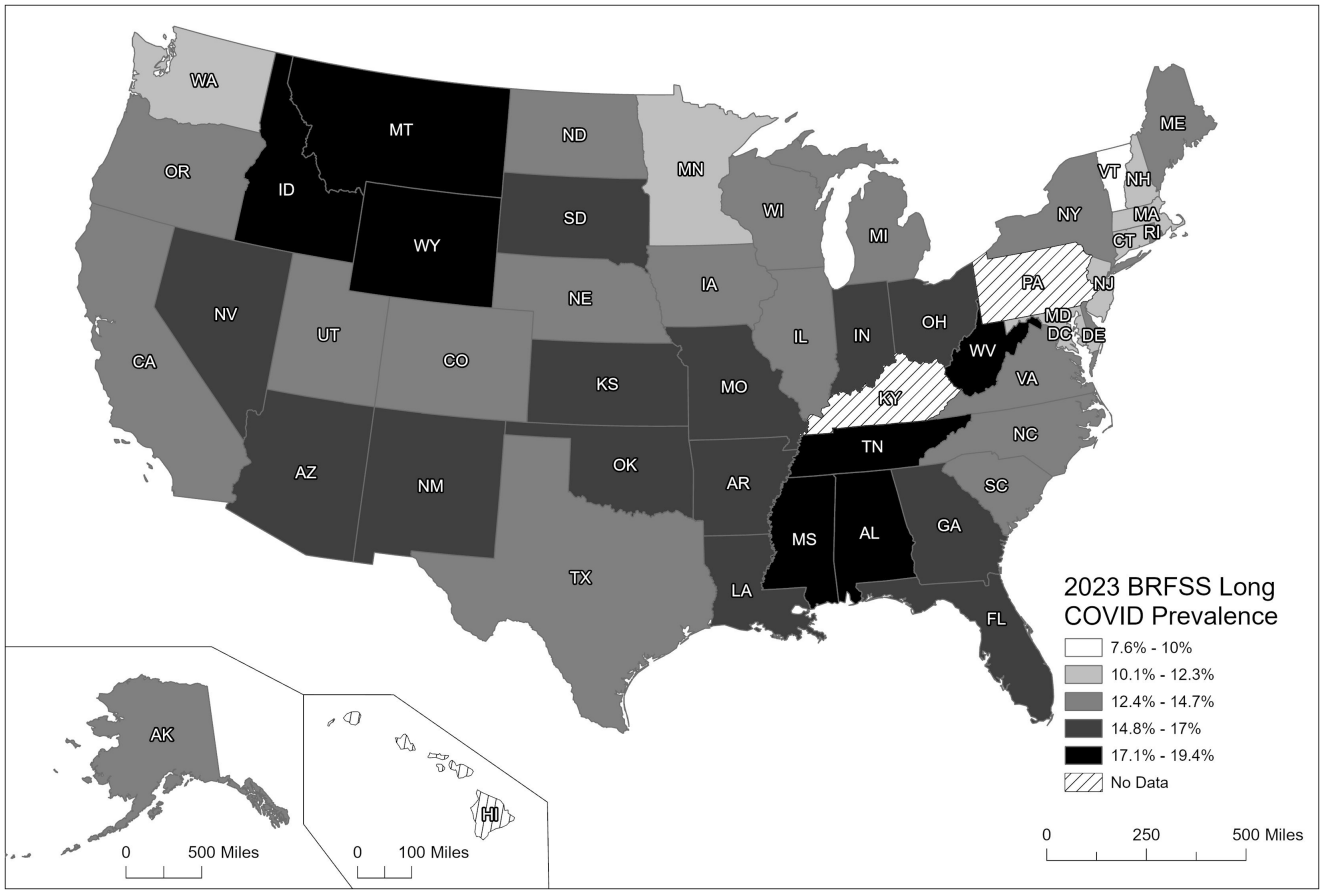
United States state- and territory-specific rates for long COVID.

Figure 1 illustrates the variability seen in the prevalence of LC across U. S. states, as reported by the 2023 BRFSS data. Parts of the South, Southwest, and lower Midwest, including Mississippi, Louisiana, Kentucky, Arkansas, and Oklahoma, recorded the highest rates of LC (15.48–19.40%). Previous studies have identified these regions of the U. S. as having higher rates of chronic conditions (e.g., obesity, type 2 diabetes, CHD, or hypertension) and health behaviors such as physical inactivity, poor dietary practices, or smoking (23, 24). In addition, these same states report lower prevalence rates for preventive practice behaviors of vaccinations for COVID-19 as well as influenza and other vaccine preventable diseases.

In contrast, states in the Upper Midwest and Northwest, including Minnesota, North Dakota, and Montana, similarly had the lowest prevalence rates of LC (7.60–11.53%). These findings are supported by lower prevalence rates of chronic conditions, behavioral risk factors, and higher rates of preventive health behaviors such as vaccinations compared to the states in the south, southeast, and mid-west, while the states in the West and Northeast indicated a moderate prevalence range of LC (11.54–15.47%). Kentucky and Pennsylvania were the only states not reporting LC because they had elected not to administer the BRFSS COVID-19 module during 2023.

Another important finding examining these state-specific estimates for LC was that 21% of respondents with LC reported having significant limitations in their activities of daily living, with another 38% of respondents with LC reporting some limitations, and the remaining proportion (41%) with LC reporting not having any significant limitations.

## Discussion

The present findings support the hypothesis that adults with confirmed COVID-19 and persisting symptoms are at increased risk of LC if they report chronic conditions such as overweight/obesity, current hypertension, current asthma, or a history of coronary heart disease; the behavioral risk factor of not meeting PAGs for either aerobic or strengthening activity; or fewer than three COVID-19 vaccinations compared with adults who received four or more vaccinations. Potential risk factors and protective factors for LC have been previously examined by a number of investigators. Luo et al. in their meta-analysis reported risk factors for LC to include being female, age 19+, severity of COVID infection, greater than one comorbidity, prolonged hospital stay, high BMI, and high basal metabolic rate (19). Crook et al. reported risk factors associated with LC that included age, with ≥50 being highest risk, and a greater number of pre-existing NCDs (20). The pre-existing NCDs included hypertension, obesity, psychiatric conditions, and immunosuppressive conditions (20). Protective factors included wearing masks, washing hands, social support, physical activity, adequate sleep, and moderate intake of alcohol (20). Similarly, a study from Catalonia examined risk factors and determinants of LC among 2,764 patients (21). The prevalence of LC was 23% among this cohort between 2021 and 2023. Sociodemographic risk factors included being female (RR = 1.47, 95% CI 1.26–1.71), lower education than university (RR = 1.31, 95% CI 1.03–1.67), and age≥65 (RR = 0.77, 95% CI 0.61–0.98). Lifestyle risk factors for long COVID included sleep duration ≤5 h (RR = 1.31 95% CI 1.06–1.62) or ≥9 h (RR = 1.74, 95% CI 1.30–2.34) and BMI ≥ 30 (RR = 1.39, 95% CI 1.20–1.62). Prior medical history risk factors for long COVID included reporting regular/poor perceived health in 2020 (RR = 2.26, 95% CI 1.89–2.71) and presence of any prior chronic condition (RR = 1.58, 95% CI 1.38–1.80). COVID-19 infection severity was another risk factor for long COVID, with mild/moderate infection having 3x higher risk (RR = 3.10, 95% CI 2.20–4.36) and those with severe/critical infection having a > 9x higher risk (RR = 9.88, 95% CI 6.88–14.18) compared to those asymptomatically infected. Protective factors in this study included vaccination prior to infection (RR = 0.33, 95% CI 0.29–0.38), vaccination within 3 months post-infection (RR = 0.58, 95% CI 0.39–0.86), and physical activity levels, with moderate to high intensity being better than low (RR = 0.80, 95% CI 0.67–0.96, high vs. low physical activity).

Relative to our findings demonstrating a strong association between being fully vaccinated with the COVID-19 vaccine and lesser odds of having LC, others with more robust clinical data have published similar findings. Kuodi et al. and his group in Israel identified patients receiving the BNT162b2 vaccination as having a lower incidence of LC compared to patients not receiving the vaccination (25). Krishna, et al. and his colleagues in monitoring the incidence of LC among their patients in Cambridge, the UK, also documented a lower incidence among those patients who received a COVID-19 vaccine series, with those not vaccinated manifesting a greater incidence of LC (26). These authors also reported that those patients with existing co-morbidities post vaccination, appeared to be slightly more susceptible to LC but still had rates below those unvaccinated with similar existing NCD co-morbidities. Ayoubkhani and colleagues provided longitudinal data to track the trajectory of signs and symptoms of LC (27). Their data provided insights into the persistence and the time course of these symptoms following COVID-19 vaccination. These investigators were able to demonstrate a significant dose–response relative to the prevalence of LC, with those patients manifesting a greater prevalence of LC following a single dose of the vaccine (mRNA) with a lower prevalence among those with a two-dose sequence. Unfortunately, the 2023 BRFSS was deficient in providing any sort of follow-up but rather a population-based cross-sectional survey. In addition, for the year 2023, the COVID-19 module did not include a listing of symptoms and/or signs from which respondents could respond. Although respondents were provided the opportunity to seek more guidance from the interviewers about symptoms, there was no indication within the 2023 BRFSS data of which respondents received this clarification. Hence, we were unable to discriminate among those LC respondents about what symptoms and organ systems were affected by their LC.

Swank et al. identified spike protein remnants among 578 out of 706 participants after their most recent COVID-19 infection. Of significance was the association between the presence of these spike proteins and documented LC among 82% of the 578 participants (28). More recent findings have shed further insights into the pathophysiology of LC that goes beyond the remnant spike proteins of the SARS-CoV-2 virus. Alhudiri et al. have documented that LC is associated with cellular and molecular disturbances which include evidence of endoplasmic reticulum stress, immune dysregulation, and oxidative imbalance (29). Nieman has recently summarized the current literature providing new insights into the role of regular physical activity and associated immunological adaptations resulting in the prevention and control of infectious disease outcomes including COVID-19 and LC (30). de Melo et al., further elucidated the impact of spike proteins and LC (31).

There then appears to be a tenable mechanism for a significant association with LC from these findings as well as others. That is, there is a potential synergism between the host’s immune response to remnant spike protein particles and other cellular and biochemical responses due to insufficient physical activity, having less than four or more COVID-19 vaccinations, the presence of the chronic conditions of coronary heart disease, hypertension, current asthma, overweight and/or obesity, and the socio-demographic variables of female sex, age less than 65 years, and educational attainment less than college or technical school completion.

Hence, LC itself may have emerged from the pandemic as a chronic disease. Management of existing NCDs and their antecedent risk factors as well as the prevention of secondary conditions could be a significant challenge to primary care providers as well as patients during an outbreak such as the recent COVID-19 pandemic (32). Such management can be further exacerbated by complications associated with prolonged effects on function and wellbeing in the context of a post-COVID-19 infection (33). Although a mild to moderate acute infection with SARS-CoV-2 usually lasts somewhere between 1 and 2 weeks, a minority of patients, albeit a significant number, manifest prolonged or resurgent symptoms of COVID-19 for a much longer period, with subacute symptoms lingering for at least 4 weeks and beyond to even years following their initial infection (5, 32). Although the prolongation of COVID-19 symptoms is often referred to as ‘LC’, this COVID-19 post-acute infection is also referred to as post-acute sequelae of COVID-19 (PASC) or post-COVID condition (32). However, since the publication of the proceedings of this LC workshop, a specific and rare type of LC in children has emerged called the multisystem inflammatory syndrome in children (MIS-C) (34). The recovery process and trajectory of recovery from LC is still being investigated and only recently have results begun to emerge from the National Institute of Health’s RECOVER study (35). Regardless, future investigations among persons suffering from LC should include follow-up and surveys to track the trajectory of recovery among such patients. The BRFSS could serve to provide an additional set of questions asking respondents who have had LC about length of time with symptoms and when such symptoms completely subsided.

The initial and most frequent site of an acute infection with SARS-CoV-2 is the respiratory system, however, the virus can invade other organ sites where angiotensin converting enzyme 2 (ACE2) receptors are located, including the heart, brain, kidney, and gut. These same sites can also be plagued by the effects of LC, with recent findings having identified an array of more than 50 persistent signs/symptoms constituting LC (36, 37).

Rates of LC appear to be associated with more severe cases and are more likely to occur among patients who required hospitalization for COVID-19 (13). In addition, LC rates have been shown to be significantly lower among individuals with healthier lifestyles, including regular physical activity, having a healthy diet, being a nonsmoker, and being fully vaccinated, prior to becoming infected with SARS-CoV-2 (38). Thus it appears that those who are more susceptible to LC have similar profiles to those who are at greater risk of developing a severe COVID-19 infection in terms of the following: (1) 65 years or older; (2) with underlying NCD (e.g., obesity, hypertension, coronary heart disease, type 2 diabetes mellitus); (3) who are physically inactive; and (4) were not vaccinated or fully vaccinated (5, 39, 40). These data support the importance of public health agencies and healthcare systems actively promoting and supporting healthy lifestyles and preventive practices as fundamental to an effective pandemic prevention effort (41–43).

Throughout the pandemic, to overcome patient hesitancy and restrictions for in-person medical appointments, the use of telemedicine and virtual health/medical visits became more common and well accepted as an alternative for health care, affording the most protection from infection for both patient and healthcare provider alike (44, 45). Such encounters continued as follow-up visits for those manifesting signs of LC. Hence, the convenience and the advancement in technology afforded through this method of healthcare delivery have evolved into a level of care that is now often considered standard. Further breakthroughs with diagnostic technology, mobile testing, and real-time health consultation experience should offer this approach as an acceptable standard of care and useful tool in the face of future pandemics and emergency response conditions (46, 47).

A number of limitations exist in conducting this study: (1) The BRFSS surveys, whose questions have been shown to be both valid and reliable, are none-the-less self-reported information and subject to respondent recall bias (21); (2) the specific questions regarding having a positive COVID-19 test may be subject to under- or over-reporting; (3) the number of COVID-19 vaccinations do not delineate between the first two doses and/or subsequent ‘boosters’; (4) the BRFSS is a cross-sectional survey, therefore associations cannot be determined for cause and effect; and (5) signs and symptoms for LC were not assessed among those respondents who indicated that they had LC. Despite these potential limitations, the study has several strengths: (1) a large sampling frame and sample size (>400,000); (2) state-specific estimates and a nationally representative sample; (3) more than 200,000 self-reported COVID-19 cases and 27,000 reported LC cases, providing a robust dataset for examining key associations with long COVID; and (4) the most recent BRFSS data including the full physical activity module, permitting estimates for PAGs as well as type, duration, frequency, and relative intensity of both aerobic and strengthening activities.

## Conclusion

The present findings demonstrate a 13.6% overall national prevalence estimate for LC across all states and territories, except for Pennsylvania and Kentucky, as they did not utilize the COVID-19 module. An important finding was that many adults with LC reported that the condition had a negative impact on their activities of daily living. The pattern of LC prevalence across the U.S. was also found to be heterogeneous, with those states in the south, southeast, and upper mid-west reporting the highest prevalence rates. Our results confirmed the associations for LC with selected NCD/chronic disorders, female sex, and lower educational attainment, as well as the health behavior of not achieving the PAG for aerobic and/or strength and the preventive practice of having fewer than three COVID-19 vaccinations. These associations support the hypothesis that meeting PAGs and being fully vaccinated may offer protection against LC following COVID-19, potentially through a synergistic effect between the immune benefits of sufficient physical activity and full vaccination (41, 42).

## Data Availability

Publicly available datasets were analyzed in this study. This data can be found here: https://www.cdc.gov/brfss/annual_data/annual_2023.html.
